# Comparison of Outcomes Between Staged and Same-Day Circumferential Spinal Fusion for Adult Spinal Deformity: Systematic Review and Meta-Analysis

**DOI:** 10.2196/67290

**Published:** 2025-03-06

**Authors:** Mert Marcel Dagli, Ryan William Turlip, Felix C Oettl, Mohamed Emara, Jaskeerat Gujral, Daksh Chauhan, Hasan S Ahmad, Gabrielle Santangelo, Connor Wathen, Yohannes Ghenbot, John D Arena, Joshua L Golubovsky, Ben J Gu, John H Shin, Jang Won Yoon, Ali K Ozturk, William C Welch

**Affiliations:** 1 Department of Neurosurgery Perelman School of Medicine University of Pennsylvania Philadelphia, PA United States; 2 Hospital for Special Surgery New York, NY United States; 3 Department of Orthopedic Surgery Balgrist University Hospital University of Zurich Zurich Switzerland; 4 College of Medicine University of Sharjah Sharjah United Arab Emirates

**Keywords:** adults, circumferential fusion, scoliosis, spinal curvature, spinal fusion, spinal deformity, intraoperative, postoperative, perioperative, systematic reviews, meta-analysis, PRISMA

## Abstract

**Background:**

Adult spinal deformity (ASD) is a prevalent condition often treated with circumferential spinal fusion (CF), which can be performed as staged or same-day procedures. However, evidence guiding the choice between these approaches is lacking.

**Objective:**

This study aims to compare patient outcomes following staged and same-day CF for ASD.

**Methods:**

Following PRISMA (Preferred Reporting Items for Systematic Reviews and Meta-Analyses) guidelines, a comprehensive literature search was conducted in PubMed, MEDLINE, Embase, Cochrane CENTRAL, Web of Science, and Scopus. Eligibility criteria included studies comparing outcomes following staged and same-day CF in adults with ASD. Searches were exported to Covidence, and records were deduplicated automatically. Title and abstract screening, full-text review, and data extraction were performed by two independent reviewers, with all conflicts being resolved by a third reviewer. A meta-analysis was conducted for outcomes reported in 3 or more studies.

**Results:**

Seven studies with 741 patients undergoing CF for ASD were included in the review (staged: n=331, 44.7% and same-day: n=410, 55.3%). Four studies that had comparable outcomes were merged for the quantitative meta-analysis and split based on observed measures. The meta-analysis revealed significantly shorter hospital length of stay (mean difference 3.98, 95% CI 2.23-5.72 days; *P*<.001) for same-day CF. Three studies compared the operative time between staged and same-day CF, with all reporting a lower mean operative time for same-day CF (mean between 291-479, SD 129 minutes) compared to staged CF (mean between 426-541, SD 124 minutes); however, inconsistent reporting of mean and SD made quantitative analyses unattainable. Of the 4 studies that compared estimated blood loss (EBL) in the relevant groups, 3 presented a lower EBL (mean between 412-1127, SD 954 mL) in same-day surgery compared to staged surgery (mean between 642, SD 550 to 1351, SD 869 mL). Both studies that reported intra- and postoperative adverse events showed more intraoperative adverse events in staged CF (10.9% and 13.6%, respectively) compared to same-day CF (9.1% and 3.6%, respectively). Four studies measuring any perioperative adverse events showed a higher incidence of adverse events in staged CF than all studies combined. However, quantitative analysis of EBL, intraoperative adverse events, and perioperative adverse events found no statistically significant difference. Postoperative adverse events, reoperation, infection rates, and readmission rates showed inconsistent findings between studies. Data quality assessment revealed a moderate degree of bias for all included studies.

**Conclusions:**

Same-day CF may offer shorter operating time and hospital stay compared to staged CF for ASD. However, there was marked heterogeneity in perioperative outcomes reporting, and continuous variables were inconsistently presented. This underscored the need for standardized reporting of clinical variables and patient-reported outcomes and higher evidence of randomized controlled trials to elucidate the clinical superiority of either approach.

**Trial Registration:**

PROSPERO CRD42022339764; https://www.crd.york.ac.uk/prospero/display_record.php?RecordID=339764

**International Registered Report Identifier (IRRID):**

RR2-10.2196/42331

## Introduction

Adult spinal deformity (ASD) is defined as abnormal curvature of the spine and is becoming increasingly prevalent, affecting up to 68% of the older adult population [1,2]. ASD is a complex spectrum of spinal pathology, including deformities such as lordosis, kyphosis, or scoliosis of the lumbar and thoracic spinal column. Although untreated adolescent ASD does occur, it typically presents in patients older than 60 years due to factors such as age-related spinal degeneration or reduced bone density [1,3].

Individuals with ASD can undergo expectant (observation alone), nonoperative, or operative therapies. At present, there is no high-quality evidence to support the decisions surgeons and patients face in treatment selection [4]. In past years, pain management and physical therapy were the preferred treatment options for ASD due to the high risk of adverse events, prolonged recovery time, and financial burden associated with surgical intervention [1,5]. If nonsurgical approaches fail to improve patients’ quality of life, surgical intervention is often considered. Multicenter retrospective cohort studies previously showed an improvement in patient-reported outcomes following the surgical management of ASD [3,6]. Indications for surgery include (1) progressive curvature of the spine with sagittal or coronal imbalance, (2) significant loss of pulmonary function caused by the misalignment and deformity, and (3) loss of function due to pain associated with spinal curvature [7-10]. These are weighted against the patient comorbidities and risks of operation [11].

Long-segment surgical management by circumferential spinal fusion (CF) has increased in popularity due to its added stability granted by both anterior and posterior fixation of the spinal column [12]. CF attempts to remedy the limitations of lateral approaches alone, such as the need for intraoperative patient repositioning, which increases operative time and puts the patient at risk for adverse events due to longer time under anesthesia [13-15]. ASD can be treated by CF in 2 primary ways: staged and same day. Staged fusions occur on 2 distinct operative days, while same-day fusions are completed within a single session. Staging is largely determined by surgeon preference and case complexity, which can cause variability in the clinical management of ASD. The preference to treat with or without staging does not necessitate a gold-standard treatment for a given case complexity but rather can depend on surgical training differences and hospital administration pressures. To our knowledge, there has not been a review of published literature on staging in CF. This systematic review and meta-analysis aims to assess and quantify the patient outcomes after staged and same-day CF for ASD to guide operative decision-making and patient selection.

## Methods

### Guidelines, Protocol, and Registration

The design and reporting of this study were supported by the following guidelines: PRISMA (Preferred Reporting Items for Systematic Reviews and Meta-Analyses; Multimedia Appendix 1) and PRISMA-P (Preferred Reporting Items for Systematic Review and Meta-Analysis Protocols) [16,17]. In accordance with PRISMA-P guidelines, the protocol of the systematic review was registered on the PROSPERO (CRD42022339764) and disseminated through *JMIR Research Protocols* (PRR1-10.2196/4233), with the protocol being published before any data were collected [12]. There were no deviations from the protocol.

### Eligibility Criteria

The Population, Intervention, Comparison, Outcome (PICO) framework was used to formulate inclusion criteria.

- Population: Adults with ASD
- Intervention: Staged CF surgery
- Comparison: Same-day CF surgery
- Outcomes: Perioperative outcomes (estimated blood loss [EBL], operative time, and length of hospital stay), adverse events, infection rates, and hospital readmissions or reoperations

Studies that do not differ in surgical timing (staged vs same day), nonhuman or adolescent patient populations, reviews, conference abstracts, single-case studies, or technical notes were excluded from the analysis. Further, only studies originally published in English were considered.

### Search Strategy

Databases explored included PubMed, MEDLINE, Embase, Cochrane CENTRAL, Web of Science, and Scopus. A literature search was conducted in accordance with the PRISMA guidelines on August 2, 2023. We used a complex search string that was modified and fitted to the unique search functions of each queried database (Multimedia Appendix 2). Additional searching through gray literature and reference lists was conducted to identify studies not initially captured by the database query.

### Data Selection and Extraction

Studies and full text were screened, data were extracted using Covidence (Veritas Health Innovation), and duplicates were automatically removed by the software [18]. Titles and abstracts were first screened independently by 2 reviewers (FCO and ME). Next, the full text of each paper was assessed by 2 reviewers (FCO and ME) to determine the eligibility of the studies. At both stages, a third reviewer (MMD) resolved any conflicts. The following data were extracted by two authors (FCO and ME) with a third (MMD) resolving any conflicts: authors; publication year; location; number of patients; age; study type; population details; surgery details; and results, including intraoperative adverse events, postoperative adverse events, postoperative infection, perioperative adverse events, hospital length of stay (LOS), intensive care unit (ICU) LOS, reoperation, readmission, and patient-reported outcomes.

### Data Quality

The ROBINS-I (Cochrane) tool was used to assess the risk of bias in the included nonrandomized studies, covering bias due to confounding variables, patient selection, classification of interventions, deviations from intended interventions, missing data, measurement of outcomes, and selection of reported results [19]. Two reviewers (FCO and JG) independently scored all domains, with a third reviewer (MMD) resolving any conflicts. Robvis was used for figure generation [20].

### Data Synthesis

Studies with comparable outcomes were merged for the quantitative meta-analysis and split based on observed measures. After conducting a qualitative evaluation, we determined that there were sufficient data to perform a meta-analysis. RevMan (version 8.4; Cochrane) using random-effects modeling was used for all quantitative analyses. Mean differences for continuous variables (surgical time, EBL, hospital LOS, and ICU LOS) and odds ratios for categorical variables (intraoperative adverse events, postoperative adverse events, postoperative infection, any adverse events, readmission, and reoperation) were the end points of the meta-analysis.

## Results

### Study Identification

**Figure 1:**
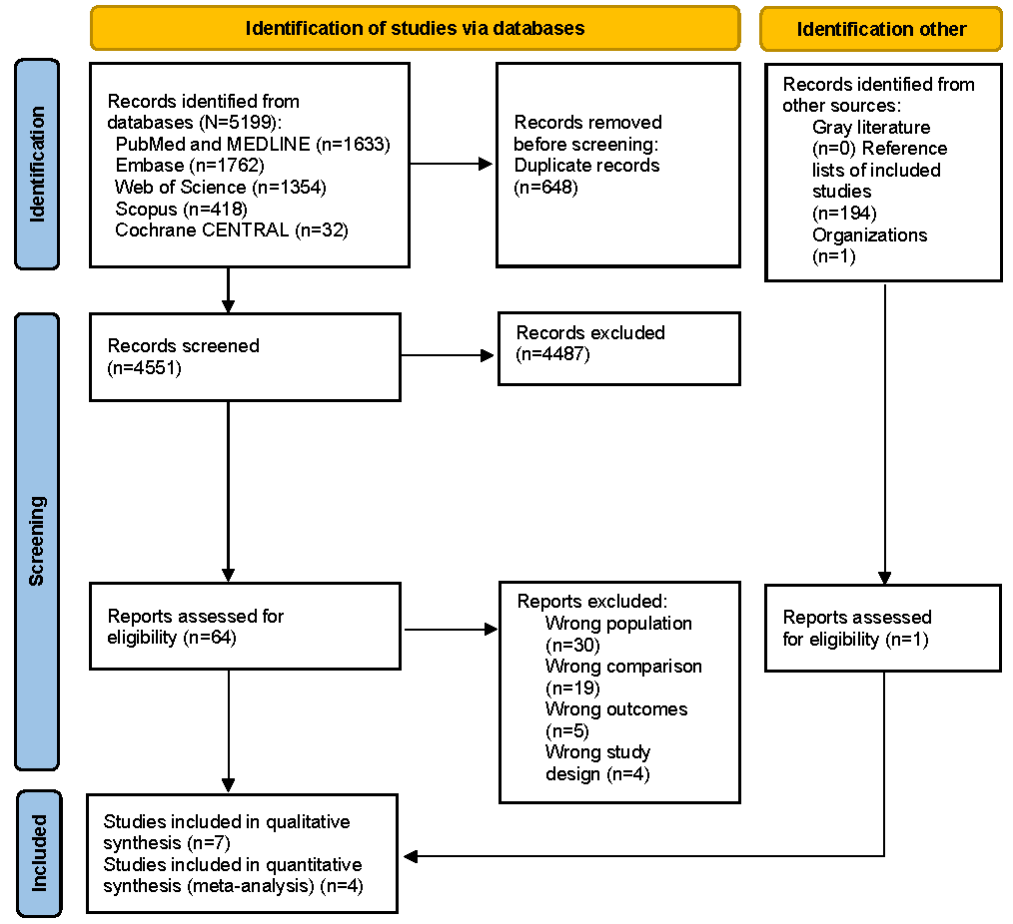
PRISMA (Preferred Reporting Items for Systematic Review and Meta-Analysis) flow diagram.

In our search (Figure 1), we identified 5199 unique studies by searching PubMed, MEDLINE, Embase, Cochrane CENTRAL, Web of Science, and Scopus, which were included for abstract screening, of which 64 were forwarded for full-text screening. After full-text review, 7 original studies were included in the data extraction process and 4 of them were in the quantitative analysis [21-27]. Studies were excluded during the full-text review for the following reasons: wrong comparator (n=19, 29.7%), wrong patient population (n=19, 29.7%), pediatric population (n=11, 17.2%), wrong outcomes reported (n=5, 7.8%), and wrong study design (n=4, 6.3%).

### Baseline Parameters

Six of the studies included were conducted in the United States [21-25,27], while 1 study was conducted in Japan [26]. The included studies describe a total of 741 patients undergoing either staged (n=331, 44.7%) or same-day (n=410, 55.3%) CF for ASD: 297 (40.1%) patients underwent anterior lumbar interbody fusion, 408 (55.1%) patients underwent lateral interbody fusion (extreme lateral interbody fusion, direct lateral interbody fusion, and lateral lumbar interbody fusion), 54 (7%) patients underwent either lateral lumbar interbody fusion or transforaminal lumbar interbody fusion, and all 741 patients underwent posterior spinal fixation. The average number of fused vertebrae across studies ranged from 4.4 to 10 (SD 3.9). The largest variation between groups within a study ranged from 7.3 (SD 3.1) in the same-day group versus 10 (SD 3.9) in the staged group. The combined mean vertebrae fused for staged and same-day CF was 7.54 (SD 2.41) and 6.62 (SD 2.40), respectively. The follow-up period over all included studies ranged from 1 to more than 36 months, and the average age of patients ranged from 58.8 (SD 9.0) to 72.3 years. The patients included in the study of Masuda et al [26] and Albayar et al [21] were inverse probability weighted to facilitate the comparison of spinal deformity and control for any differences between the groups (Table 1).

**Table 1:** Patient demographics and significant results of included studies.

Authors (year)	Country	Patients	Age	Study type	Population details and differences	Surgery details	Results
Albayar et al (2023) [21]	United States	n=44 n=56	Mean (SD): 58.8 (9.0) years Mean (SD): 62.0 (11.9) years	Retrospective cohort study, inverse probability weighted	Patients aged >18 years at the time of surgery and diagnoses of ASDa undergoing (ALIFb), and open posterior lumbar or thoracolumbar (PSFc)	Staged ALIF, and open posterior lumbar or thoracolumbar PSF, post. Vertebrae fused: mean 10 (SD 3.9) Same-day ALIF, and open posterior lumbar or thoracolumbar PSF, post. Vertebrae fused: mean 7.3 (SD 3.1)	Staged: EBLd mean 1351.7 (SD 869) mL, LOSe mean 10.5 (SD 5) days, IOAEf (n=6), POAEg (n=30), reoperation (n=10), POIh (n=5), and readmission (n=10). Same day: EBL mean 1127.6 (SD 945.4), LOS mean 6.2 (SD 3.1) days, IOAE (n=2), POAE (n=30), reoperation (n=8), POI (n=1), and readmission (n=8).
Anand et al (2014) [22]	United States	n=37 n=13	Mean (range): 61 (20-85) years	Retrospective cohort study	Patients with adult idiopathic scoliosis corrections undergoing cMISSi, Cobb angle of greater than 30 but less than 75 degrees	Staged DLIFj and L5-S1 XLIFk followed by PSF. Vertebrae fused: mean 7 (range 4-15) Same-day DLIF and L5-S1 XLIF followed by PSF. Vertebrae fused: mean 7 (range 4-15)	Staged: EBL mean 763 (range 25-2500) mL and ORl time mean 482 (range 83-546) minutes. Same day: EBL mean 613 (range 150-1500) ml and OR time mean 351 (range 176-510) minutes.
Anand et al (2013) [23]	United States	n=36 n=35	Mean (range): 64 (20-84) years	Retrospective cohort study	Adults with scoliosis undergoing cMISS, two or more levels	Stage combination of DLIF and XLIF with PSF. Vertebrae fused: mean 4.4 Same-day combination of DLIF and XLIF with PSF. Vertebrae fused: mean 4.4	Staged: EBL 671 mL and OR time 426 minutes. Same day: EBL 412 mL and OR time 291 minutes.
Arzeno et al (2019) [24]	United States	n=45 n=47	Mean (95% CI): 68 (61-78) years Mean (95% CI): 68 (62-72) years	Retrospective cohort study	Patients with ASD, undergoing anterior (including lateral and anterolateral approaches) and PSF of at least 5 levels Groups differ in approach, Ponte osteotomy, 3-column osteotomy, O-arm, neuromonitoring, decompression, number of posterior levels, fused; number. of osteotomy levels, mean; and number of decompression levels	Staged circumferential spinal fusion (anterior, posterior), Ponte osteotomy (n=39), 3-column osteotomy (n=7), and decompression (n=34). Vertebrae fused: mean 8 (95% CI 5-9) Same-day circumferential spinal fusion (anterior, posterior), Ponte osteotomy (n=24), 3-column osteotomy (n=1), and decompression (n=16). Vertebrae fused: mean 9 (95% CI 9-9)	Staged: LOS mean 9 days, reoperation (n=5), readmission (n=1), POI (n=2), and PEAEm (n=12). Same day: LOS mean 6 days, reoperation (n=7), readmission (n=6), POI (n=3), and PEAE (n=7).
Harris et al (2021) [25]	United States	n=41 n=46	Mean (SD): 62.0 (8.3) years Mean (SD): 60.0 (13.0) years	Retrospective cohort study	Patients with ASD who underwent long PSF (more than 5 levels fused, with fusion to the pelvis) Groups differ in previous spine surgery, scoliosis or kyphosis, pseudarthrosis, and pelvic incidence	Staged circumferential -ALIF and PSF. Vertebrae fused: mean 8.7 (SD 0.48) Same-day circumferential – ALIF and PSF. Vertebrae fused: mean 7.4 (SD 2.4)	Staged: ODIn mean 45 (SD 17) and SRS-22ro mean 2.8 (SD 0.6) Same day: ODI mean 48 (SD 15) and SRS-22r mean 2.8 (SD 0.6)
Masuda et al (2023) [26]	Japan	n=101 n=186	Mean (SD): 72.2 (7.3) years Mean (SD): 72.4 (8.0) years	Retrospective cohort study, propensity score weighted	Patients with ASD, ≥4 fused levels and at least 1 level using LLIFp, and presence of at least 1 spinal deformity marker: scoliosis Cobb angle≥20°, sagittal vertical axis≥5 cm, pelvic tilt≥25°, pelvic incidence minus lumbar lordosis angle≥10°, and thoracic kyphosis≥60°	Staged circumferential-LLIF and PSF. Vertebrae fused: mean 7.7 (SD 2.3) Same day circumferential-LLIF and PSF. Vertebrae fused: mean 6.2 (SD 2.4)	Staged: EBL mean 642.5 (SD 550.5) mL, OR time mean 541.3 (SD 124.1) minutes, LOS mean 42 (SD 25) days, IOAE (n=11), POAE (n=11), reoperation (n=11), POI (n=4), and AAE^q^ (n=22). Same day: EBL 722.2 (SD 612.6) mL, OR time mean 479.9 (SD 128.5) minutes, LOS mean 34.1 (SD 18.2) days, IOAE (n=17), POAE (n=23), reoperation (n=19), POI (n=5), and AAE (n=40).
Than et al (2019) [27]	United States	n=27 n=27	N/Ar N/A	Retrospective cohort study	Patients with ASD, coronal Cobb angle >20, SVAs >5 cm, pelvic tilt >20, pelvic incidence–LLt >10, and thoracic kyphosis >60.	Staged MISu LLIF or MIS TLIFv with PSF. Vertebrae fused: mean 5.4 Same-day MIS LLIF or MIS TLIF with PSF. Vertebrae fused: mean 5.3	Staged: Reoperation (n=4), POI (n=0), and PEAE (n=9). Staged: Reoperation (n=7), readmission (n=1), POI (n=1), and PEAE (n=8).

^a^ASD: adult spinal deformity.

^b^ALIF: anterior lumbar interbody fusion.

^c^PSF: posterior spinal fusion.

^d^EBL: estimated blood loss.

^e^LOS: length of stay.

^f^IOAE: intraoperative adverse events

^g^POAE: postoperative adverse events.

^h^POI: postoperative infection.

^i^cMISS: circumferential minimally invasive spinal surgery.

^j^DLIF: direct lateral interbody fusion.

^k^XLIF: extreme lateral interbody fusion.

^l^OR: operating room.

^m^PEAE: perioperative adverse events.

^n^ODI: Oswestry Disability Index.

^o^SRS-22r: Scoliosis Research Society-22 revised.

^p^LLIF: lateral lumbar interbody fusion.

^q^AAE: any adverse events.

^r^N/A: not available.

^s^SVA: sagittal vertical axis.

^t^LL: lumbar lordosis.

^u^MIS: minimally invasive surgery.

^v^TLIF: transforaminal lumbar interbody fusion.

### Quantitative Analysis

**Figure 2:**
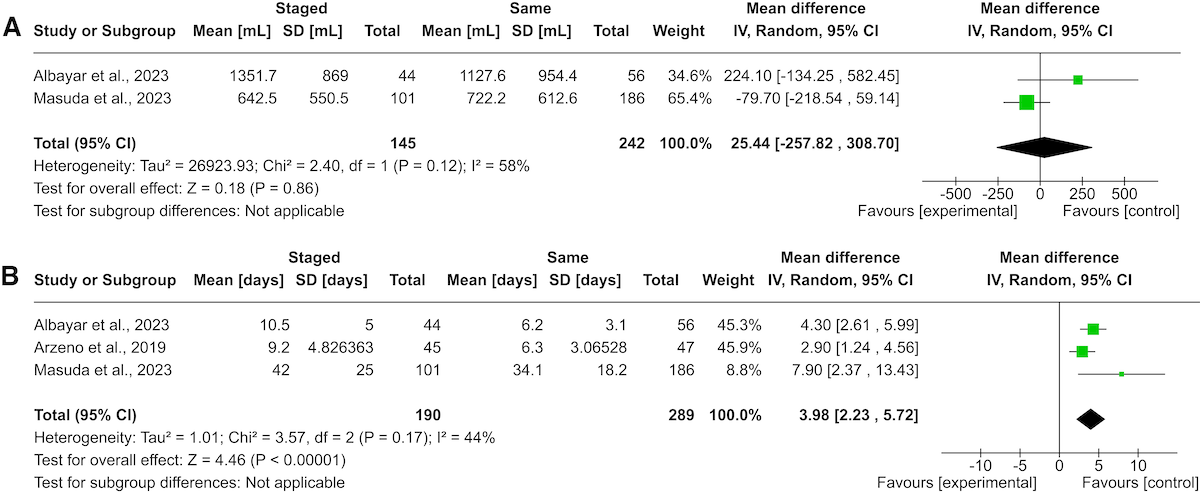
Forest plots comparing (A) estimated blood loss and (B) hospital length of stay between patients who underwent staged or same-day circumferential spinal fusion for adult spinal deformity. IV: inverse variance.

Of the included studies, 4 studies compared EBL in the relevant groups, with 3 studies presenting a lower EBL (mean between 412-1127, SD 954 mL) in same-day surgery compared to staged surgery (mean between 642, SD 550, and 1351, SD 869 mL) [21-24]. The meta-analysis shows a nonsignificant advantage for same-day surgery **(**Figure 2A [21,24,26]). Only 2 studies that measured EBL were included in the quantitative analysis because of inconsistencies in reporting, where some did not report variables as measures of variance, which made pooling in these instances not feasible. Three studies compared the operative time between staged and same-day CF, with all of them reporting a lower mean operative time for same-day CF (mean between 291-479 minutes) compared to staged CF (mean between 426-541 minutes) [22-24]. Just 1 group reported mean and SD for odds ratio time, thus restricting the potential for a quantitative analysis.

Three studies comparatively evaluated the hospital LOS [21,22,25]. All three studies consistently found that the mean LOS was less for same-day CF (mean between 6-34.1 days) in comparison to staged CF (mean between 9-42 days). The meta-analysis clearly presented a shorter LOS in patients undergoing same-day CF compared to staged CF (Figure 2B).

**Figure 3:**
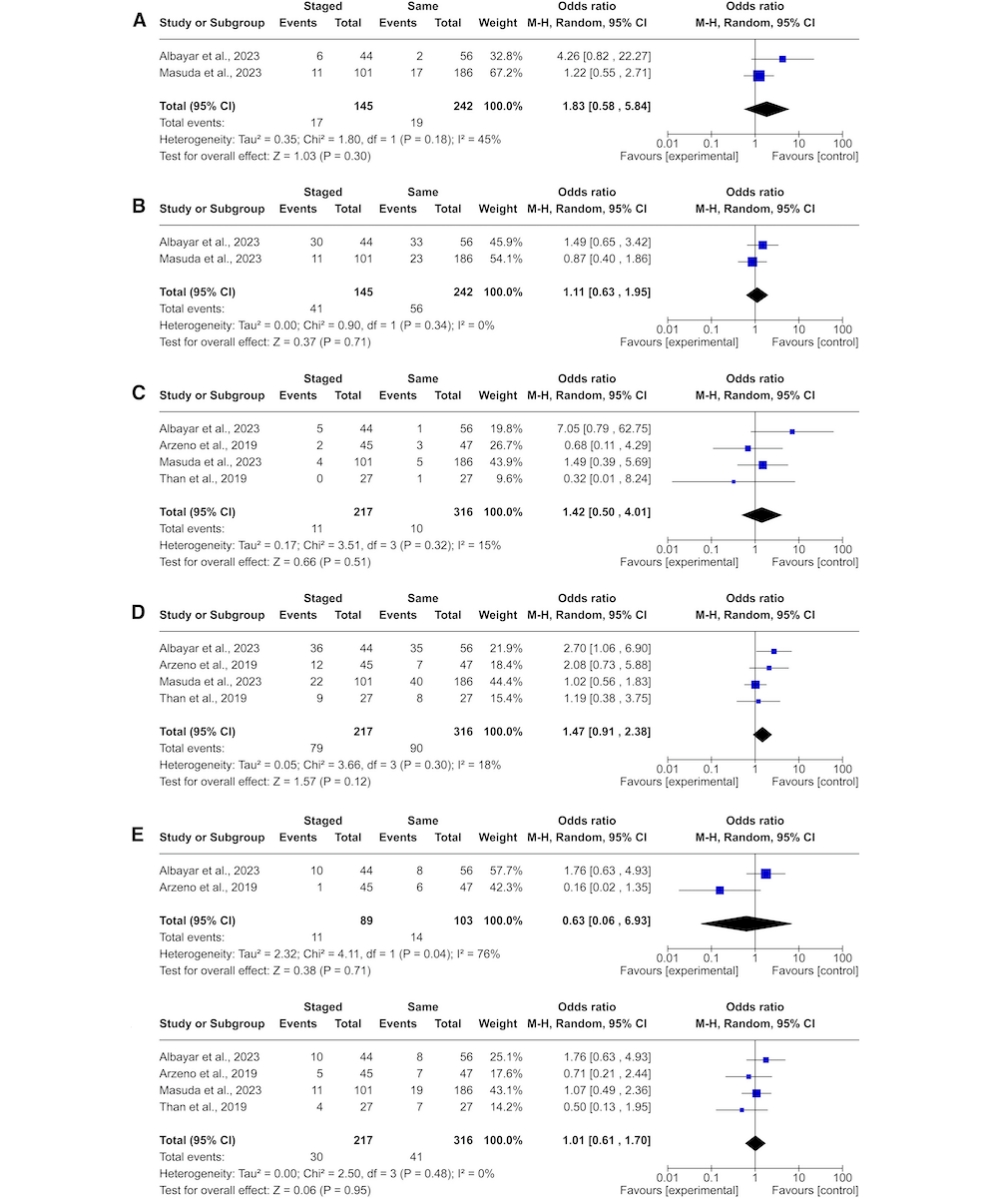
Forest plots comparing (A) intraoperative adverse event, (B) postoperative adverse event, (C) postoperative infection, (D) perioperative adverse event, (E) 30-day readmission, and (F) reoperation rates between patients who underwent staged or same-day circumferential spinal fusion for adult spinal deformity. M-H: Mantel-Haenszel.

Two of the 7 studies compared intraoperative and postoperative adverse events between staged and same-day CF procedures [21,26]. Both Masuda et al [26] and Albayar et al [21] reported more intraoperative adverse events in staged CF (10.9% and 13.6%, respectively) compared to same-day CF (9.1% and 3.6%, respectively); however, the meta-analysis failed to show a statistically significant difference between the groups (Figure 3A [21-27]). Masuda et al [26] reported fewer postoperative adverse events in staged CF (10.9 vs 12.4%), while Albayar et al [21] presented a lower incidence in same-day CF (53.6 vs 68.2%), without a significant difference in the meta-analysis (Figure 3B). Four studies measured any perioperative adverse events [21,22,26,27]. The overarching analysis showed a higher incidence of adverse events in Staged CF over all studies; however, the meta-analysis did not show significance (Figure 3D).

Four of the included studies compared reoperation and postoperative infection rates between patient groups [21,22,26,27]. Albayar et al [21] and Masuda et al [26] showed a slightly lower postoperative infection in same-day CF versus staged CF (4.4 vs 6.4%, 2.6 vs 4%, and 1.7 vs 11.4% respectively), while Than et al [27] showed a lower postoperative infection in staged CF (0% vs 3.7%**;** Figure 3C). While Arzeno et al [24] and Than et al [27] reported a lower reoperation in patients undergoing staged CF (11.1 vs 14.9% and 14.8 vs 25.9%, respectively). Masuda et al [26] and Albayar et al [21] reported less reoperation in patients undergoing same-day CF versus staged CF (10.2 vs 10.9% and 14.2 vs 22.7% respectively). However, none of these differences in reoperation reached statistical significance in either the original respective studies or our meta-analysis (Figure 3F).

Readmission rates reported by Arzeno et al [24] and Albayar et al [21] demonstrated diverging results. Arzeno et al [24] found a lower readmission for staged CF versus same-day CF (2.2 vs 12.8%), while Albayar et al [21] reported a higher readmission in staged CF versus same-day CF (22.7 vs 14.3%). The meta-analysis did not indicate a conclusive result (Figure 3E). Harris et al [25] are the only authors reporting on either the Oswestry Disability Index (ODI) or the Scoliosis Research Society Score, showing a better outcome measured by the ODI in same-day CF and no difference in Scoliosis Research Society Score.

### Risk of Bias Analysis

**Figure 4:**
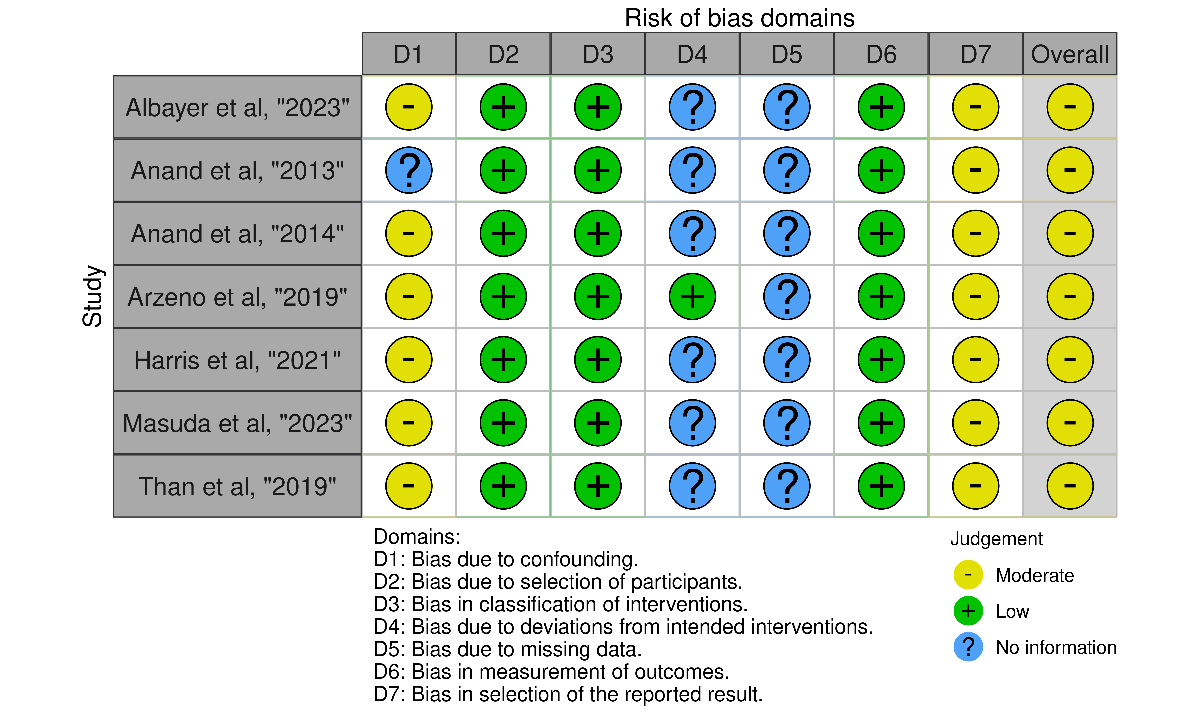
ROBINS-I traffic light plot of included studies.

There was a moderate degree of bias for all included studies (Figure 4 [21-27]).

## Discussion

### Overview

In this systematic review and meta-analysis, we compared differences in perioperative outcome variables for individuals undergoing staged or same-day CF in 7 included studies. The meta-analysis revealed a statistically significant increase in hospital LOS in the staged group when compared to the same-day group. There were no statistically significant differences in EBL, intraoperative adverse event, postoperative adverse event, postoperative infection, perioperative adverse event, 30-day readmission, and reoperation rates between patients who underwent staged or same-day CF for ASD. There were marked differences between patient populations and the subsequent clinical outcomes that were reported in each observational study, which most did not adjust for. Our quantitative results are paradoxical as there were inconsistencies between studies in the staged and same-day subgroups for certain reported outcomes, while other findings had stronger conclusive evidence. Due to the apparent differences in consensus, the research included in the following discussion is organized according to the consistency of results.

### Mixed Findings

Heterogeneity in meta-analyses grants the opportunity to examine variable factors that may be leading to the results. There were several reported outcomes that yielded inconclusive evidence in this study. Adverse event rates were reported by authors in several ways, with some splitting between intraoperative or postoperative adverse events, while others reported any adverse event throughout the perioperative course. Two studies reported more intraoperative adverse events in staged CF, but the meta-analysis failed to show a statistically significant difference. This can indicate that there is an increased operative risk when staging, but it can also be due to the generally sicker patients with more spinal deformity that is leading to increased intraoperative adverse events. Further, 1 study presented a lower postoperative adverse events rate during staged CF, while a different group found a higher rate in staged, indicating no difference in postoperative complications between the 2 approaches. When all perioperative adverse events were instead considered, every study reported a higher incidence in staged CF, and this result trended toward significance. Vastly different conclusions depending on the stage of surgery in which the adverse events are considered illuminate the need for greater consistency in reporting outcomes. However, the increased perioperative adverse events seen in staged CF, although not statistically significant, could be clinically important in decision-making.

The Centers for Medicare & Medicaid Services targeted 30-day readmission rates as a source of unnecessary costs [28]. A study conducted by McCarthy et al [29] found the total hospital costs to surgically treat ASD averaged US $120,394, with primary surgery averaging US $103,143, and total readmission costs of US $67,262 for their cohort. The high costs associated with spine surgery make patient readmission and reoperation lucrative targets. There is profound heterogeneity in reoperation and readmission rates that depend on several factors, such as patient demographics, procedure types, and institutional factors. A recent systematic review found the 30-day readmission rate in spine surgery to be 4.2% and 7.4% [30]. In this review, 2 studies reported readmission with diverging results. Arzeno et al [24] found a lower readmission for staged CF, while Albayar et al [21] reported a higher readmission for same-day CF. Likewise, 4 studies reported reoperation with a split in consensus [21,24,26,27]. Given the small number of studies that measured reoperation and readmission, the results from our meta-analysis were inconclusive. The lack of statistically significant differences in the quantitative analysis combined with the heterogeneity in the qualitative review indicates that no differences in readmission and reoperation rates exist between the 2 groups.

It was also uncommon for the included studies to report specific reasons that reoperation or readmission occurred. As readmission depends on many variables including patient comorbidities, initial risk, or operative adverse events, it is crucial for authors to include these measures in future comparative studies for stronger subgroup analyses. Postoperative infection is also a driver leading to reoperation or readmission, as well as additional health care costs [31]. A recent meta-analysis found the pooled incidence of surgical site infection in 22,475 patients to be 3.1% [32]. Our included studies also reported a low incidence of postoperative infection; however, there were inconsistencies as 3 out of the 4 groups found a lower postoperative infection in same-day CF [21,24,26,27]. There are several risk factors for postoperative infection that may lead to heterogeneity. Farshad et al [33] found that intraoperative EBL was a risk factor for postoperative infection. Some studies found BMIs to be predictors of postoperative infection, although this result remains controversial [34-36]. The discrepancies seen in this review may stem from variations in surgical techniques, perioperative antibiotic prophylaxis, institutional differences, and patient-specific risk factors. The results of this study’s meta-analysis do not support any difference between the 2 techniques in postoperation infection risk.

### Associations of Operative Variables in CF Staging

For highly invasive spine surgeries, such as long-segmented CF for ASD, patients may require extended resource use [37]. Surgical operative time and LOS are critical metrics in evaluating the efficiency and resource use of staging in CF. Shorter surgical times are generally associated with reduced intraoperative adverse events, lower anesthesia-related risks, and decreased blood loss, thus contributing to improved patient safety and outcomes [15,38]. Peng et al [39] conducted a meta-analysis on correlations between operative time and postoperative infection, and they concluded that there was a 4-fold increase in postoperative infection risk for operations greater than 3 hours [39]. The studies included in our review all found a lower mean operative time for same-day CF.

A shorter LOS is also desirable as it minimizes health care resource use and lowers costs [40]. Further, early mobilization and discharge are associated with improved patient satisfaction and reduced psychological stress [41]. However, reducing LOS has also been associated with an increasing readmission rate at the population level [42]. Our meta-analysis found a significantly lower LOS for same-day CF compared to staged CF. This difference in LOS may be attributed to the cumulative effect of multiple hospital admissions, prolonged recovery periods between surgeries, and the need for additional preoperative preparation in staged CF. While the lower operative time and LOS results in patients undergoing same-day CF seem intuitive, it is important to interpret them cautiously, considering potential factors such as patient selection, discharge criteria, and institutional practices that may differ between studies. Moreover, while shorter operative time and LOS may reduce health care costs and improve resource allocation, they should not compromise patient safety or postoperative care quality.

The results of shorter LOS and operative time combined with the lack of conclusive differences between groups for adverse events, readmission, reoperation, and infection rates indicate that patient prognosis is similar when between staging preferences. The shorter time spent in the hospital and operating room is clinically important and contributes to improved patient safety and outcomes while reducing hospital resource use. However, the potential advantages of staging may outweigh these benefits and thus improve patient prognosis for more complex cases with a higher degree of relative deformity. Therefore, the results from this review and meta-analysis show preference toward same-day CF, while staging may largely depend on a patient’s condition. Further studies investigating the impact of patient-specific factors, for example, varying degrees of deformity severity, on treatment selection and subsequent outcomes are required.

### Limitations

There are several limitations to this study and the papers included in this review. First, the limited literature comparing staged and same-day CF resulted in only 7 studies being included, which reduced the statistical power of the meta-analysis as only 4 studies qualified for quantitative analysis. All the included studies are retrospective cohort studies with a level of evidence of III or II, and only 2 studies used inverse probability weighting to control for between-patient differences. Consequently, there was a moderate potential for bias, which limited our ability to reach generalizable conclusions. To elucidate the differences in surgical treatment options for CF, it will be necessary to investigate staging in level I or II randomized controlled trials (RCTs). Without RCTs, it is difficult to adjust for surgeon preference bias and the complexity of the case. Each study also demonstrated heterogeneity with respect to the patient outcomes reported, further limiting the ability for robust statistical analysis. Continuous variables were generally poorly reported since many did not report measures of variance such as mean and SD, but rather mean or median and range (min-max or IQR). This made pooling in those instances not feasible, limiting the generalizability of results. Further, only 1 group presented patient-reported outcomes, such as ODI, which is a standardized clinical variable that can be useful for measuring subjective pain and disability. Novel studies should aim to investigate patient-reported outcomes and standardize the variables being reported, so future meta-analyses will have wider samples to yield more conclusive evidence for staging differences in CF. In doing so, the mixed results presented in this study can be further assessed to support wider implementation of either staged or same-day CF while integrating clinically significant metrics to evaluate patient outcomes.

### Conclusions

Here, we present the first systematic review and quantitative meta-analysis on staging in CF to treat patients with ASD. Operative time and hospital LOS were significantly lower in same-day CF surgery, with EBL and perioperative adverse events also trending toward significance. However, there was heterogeneity in additional operative measures, such as intra- or postoperative adverse event rates, reoperation, and readmission, and no differences were found in our meta-analysis. Based on our results, it is suggested that same-day CF is advantageous as a potential time-save with patients spending less time in the operating room and hospital, potentially saving costs. However, it is still unclear whether either same-day or staged CF provides a clinical advantage for patient outcomes. Additional level I and II RCTs should be conducted to elucidate the associations between these variables and provide stronger evidence in favor of either approach.

## Appendix

Multimedia Appendix 1 PRISMA (Preferred Reporting Items for Systematic Reviews and Meta-Analyses) checklist.

Multimedia Appendix 2 Supplemental search strings for query.

## Abbreviations

ASD: adult spinal deformity
CF: circumferential spinal fusion
EBL: estimated blood loss
ICU: intensive care unit
LOS: length of stay
ODI: Oswestry Disability Index
PICO: Population, Intervention, Comparison, Outcome
PRISMA: Preferred Reporting Items for Systematic Reviews and Meta-Analyses
PRISMA-P: Preferred Reporting Items for Systematic Review and Meta-Analysis Protocols
RCT: randomized controlled trial

## Data Availability

All data generated or analyzed during this study are included in this published paper.
