# Supplementary material for: Comparison of Outcomes Between Staged and Same-Day Circumferential Spinal Fusion for Adult Spinal Deformity: Systematic Review and Meta-Analysis
Source: Interact J Med Res. 2025 Mar 6;14:e67290. doi: 10.2196/67290 (PMC11926459; doi:10.2196/67290)
Supplement: Multimedia Appendix 2 [file ijmr_v14i1e67290_app2.docx]

MEDLINE - 1,633

Embase - 1,762

Cochrane CENTRAL - 32

Web of Science - 1,354

Scopus - 418

MEDLINE

- Search #1: (“spinal curvatures”[MeSH Terms] OR “spinal curvatures”[MeSH Terms] OR “adult spinal deformity”[tiab] OR “adult degenerative deformity”[tiab] OR “asd”[tiab] OR “spinal deformity”[tiab])

- Search #2: (“staging”[tiab] OR “staged”[tiab] OR “same day”[tiab] OR “stag*”[tiab])

- Search #3: (“circumferential”[tiab] OR “anterior posterior”[tiab] OR (“anterior”[tiab] AND “posterior”[tiab]) OR “posterior”[tiab] OR “anterior”[tiab])

- Search #4: (“fusion”[tiab] OR “spinal fusion”[tiab] OR “spinal surgery”[tiab] OR “spinal fusion surgery”[tiab])

- (#1 AND #2) OR (#2 AND #3 AND #4)

- Full search: ((“spinal curvatures”[MeSH Terms] OR “spinal curvatures”[MeSH Terms] OR “adult spinal deformity”[tiab] OR “adult degenerative deformity”[tiab] OR “asd”[tiab] OR “spinal deformity”[tiab]) AND (“staging”[tiab] OR “staged”[tiab] OR “same day”[tiab] OR “stag*”[tiab])) OR ((“staging”[tiab] OR “staged”[tiab] OR “same day”[tiab] OR “stag*”[tiab]) AND (“circumferential”[tiab] OR “anterior posterior”[tiab] OR (“anterior”[tiab] AND “posterior”[tiab]) OR “posterior”[tiab] OR “anterior”[tiab]) AND (“fusion”[tiab] OR “spinal fusion”[tiab] OR “spinal surgery”[tiab] OR “spinal fusion surgery”[tiab]))

Embase

- Search #1: (('spine disease'/exp OR 'spine disease") AND stag*)

- Search #2: ('article'/it)

- Search #3: ('human'/de)

- Search #4: ('adolescent' OR 'child' OR 'infant' OR 'newborn' OR 'embryo' OR 'fetus’)

- Search #5: ('neoplasm' OR 'fracture' OR 'infection' OR 'spine metastasis' OR 'parkinson disease' OR 'heart atrium septum defect' OR 'autism' OR 'tuberculosis' OR 'case report' OR 'gene expression' OR 'cadaver' OR 'spine tumor')

- **(#1 AND #2 AND #3) NOT (#4 OR #5)**

- Full search: ('spine disease'/exp OR 'spine disease') AND stag* AND 'article'/it AND 'human'/de NOT ('adolescent' OR 'child' OR 'infant' OR 'newborn' OR 'embryo' OR 'fetus' OR 'neoplasm' OR 'fracture' OR 'infection' OR 'spine metastasis' OR 'parkinson disease' OR 'heart atrium septum defect' OR 'autism' OR 'tuberculosis' OR 'case report' OR 'gene expression' OR 'cadaver' OR 'spine tumor')

Cochrane Central Register of Controlled Trials

- Search #1: MeSH descriptor: [Spinal Curvatures] explode all trees

- Search #2: (stag*):ti,ab,kw

- #1 AND #2

Web of Science

- Search #1: (ALL=(stag*))

- Search #2: (ALL=(adult spinal deformity OR adult degenerative deformity OR adult deformity OR scoliosis OR kyphosis OR lordosis OR spinal deformity OR degenerative deformity OR ASD OR deformity OR degenerative OR kyphoscoliosis))

- Search #3: (ALL=(adult) AND DT=(Article) AND LA=(English))

- #1 AND #2 AND #3

- Full search: (ALL=(stag*)) AND (ALL=(adult spinal deformity OR adult degenerative deformity OR adult deformity OR scoliosis OR kyphosis OR lordosis OR spinal deformity OR degenerative deformity OR ASD OR deformity OR degenerative OR kyphoscoliosis)) AND (ALL=(adult) AND DT=(Article) AND LA=(English))

Scopus

- Search #1: TITLE-ABS-KEY(stag*)

- Search #2: TITLE-ABS-KEY(adult spinal deformity OR adult degenerative deformity OR adult deformity OR scoliosis OR kyphosis OR lordosis OR spinal deformity OR degenerative deformity OR ASD OR deformity OR degenerative OR kyphoscoliosis)

- Search #3: TITLE-ABS-KEY(adult)

- #1 AND #2 AND #3
